# Nonsurgical Outpatient Therapies for the Management of Female Stress Urinary Incontinence: Long-Term Effectiveness and Durability

**DOI:** 10.1155/2011/176498

**Published:** 2011-06-23

**Authors:** G. Willy Davila

**Affiliations:** Section of Urogynecology, Department of Gynecology, Cleveland Clinic Florida, 2950 Cleveland Clinic Boulevard, Weston, FL 33331, USA

## Abstract

*Objective*. To evaluate long-term effectiveness and safety of conservative and minimally invasive outpatient treatments for female stress urinary incontinence (SUI) through a review of the literature. *Methods*. PubMed was searched for reports on prospective clinical trials with at least 12-month follow-up of minimally invasive treatments, pelvic floor rehabilitation, or pharmacotherapy in women with SUI. Each report was examined for long-term rates of effectiveness and safety. *Results*. Thirty-two clinical trial reports were included. Prospective long-term studies of pelvic floor rehabilitation were limited but indicated significant improvements with treatment adherence for at least 12 months. Poor initial tolerability with duloxetine resulted in substantial discontinuation. Most patients receiving transurethral radiofrequency collagen denaturation or urethral bulking agents reported significant long-term improvements, generally good tolerability, and safety. *Conclusions*. Conservative therapy is an appropriate initial approach for female SUI, but if therapy fails, radiofrequency collagen denaturation or bulking agents may be an attractive intermediate management step or alternative to surgery.

## 1. Introduction

Female patients with stress urinary incontinence (SUI) typically present with a complaint of involuntary urine leakage upon coughing or sneezing, exertion, or with changes in posture [1, 2]. This condition is caused by weakening of the pelvic floor muscles that support the bladder and urethra (e.g., urethral hypermobility) and/or by weakness of the urethral sphincter (i.e., intrinsic sphincter deficiency [ISD]) [3]. Prevalence estimates vary widely, ranging from 4% to 35% of women [2, 4]. Aging, obesity, white race, pregnancy and vaginal childbirth, vaginal or pelvic surgery, and smoking are well-established risk factors for the development of SUI [3, 5]. Although many women do not immediately seek treatment for SUI symptoms [3, 6], the negative impact of SUI on daily functioning, sexual relationships, and overall quality of life [4, 7] are factors that can eventually prompt women to discuss treatment options with their health care providers.

Widely used for first-line treatment, pelvic floor rehabilitation primarily comprises pelvic floor muscle training (PFMT), also known as Kegel exercises, and may include pelvic muscle electrical stimulation, biofeedback, or the use of physical devices, such as vaginal cones or pessaries [8–10]. Typically when a conservative therapy fails, pharmacotherapy may be considered as a next management step. However, while the selective norepinephrine reuptake inhibitor duloxetine is approved for treatment of SUI in many countries, no pharmacologic treatments are approved in the US [5, 9]. Historically, when pelvic floor rehabilitation fails, surgical intervention remains a popular treatment option for women with moderate to severe SUI. Retropubic suspension and sling procedures are associated with 6-month short-term cure rates as high as 85% to 90% [3], with one long-term retrospective study reporting a cure rate plateau of about 69% at 10 to 12 years postsurgery [11]. While less invasive surgical options known as minislings were recently approved, they appear to be less effective than traditional slings [12–14] and pose the risk of rare but serious complications, most frequently erosion through the vaginal epithelium [3, 15, 16].

Surgery of any type, however, is not suitable for all patients. In particular, women with significant comorbidities may not be good surgical candidates. Moreover, some otherwise healthy SUI patients may find unacceptable the associated risks, pain, and recovery time, with no exertional activities, lifting more than 5 to 10 pounds, and sexual activity for 6 weeks, and choose to forgo surgery, despite continuing SUI symptoms. 

As an alternative to surgery, minimally invasive nonsurgical procedures for the management of SUI have become available. These include transurethral radiofrequency collagen denaturation, which is indicated only for patients with SUI due to urethral hypermobility, and the injection of bulking agents, which is indicated only for patients with SUI due to ISD. Results from shorter-term, randomized clinical trials have demonstrated efficacy with these therapies (versus no treatment or baseline) [17, 18]. Both types of procedure appear to be generally well tolerated [17, 19] in the respective patient populations, and neither appears to limit the possibility of later surgery. In light of their growing use, it is necessary to determine how each of these options compares with established conservative approaches with regard to long-term durability of benefit. 

This paper aims to evaluate available evidence concerning long-term (12 months or longer) efficacy of minimally invasive treatments for female SUI. To establish a baseline for comparison, long-term results with pelvic floor rehabilitation and pharmacotherapy were also included. The literature was searched to identify prospective clinical trials conducted in women with SUI with a minimum study duration of 12 months. 

## 2. Methods

### 2.1. Literature Search Strategies

PubMed searches were conducted in January 2010 and were limited to papers on clinical or randomized controlled trials conducted in humans and published in the English language. Patient populations were limited to adult females. No restrictions with regard to the country of publication were applied to any search, nor were publication dates limited to any specific time periods. The primary terms used in all searches were “urinary incontinence OR urinary stress incontinence OR urinary incontinence, stress.” These terms were combined with specific additional terms in boolean searches to locate articles on conservative therapies, pharmacotherapy, transurethral radiofrequency collagen denaturation, and bulking agents used for treatment of SUI. 

To identify articles on studies of pelvic floor rehabilitation, secondary terms used in the boolean search included “physiotherapy OR pelvic floor muscle training OR Kegel OR muscle OR electrical stimulation OR pessaries OR pessary OR biofeedback OR biostimulation OR cone OR vaginal cone OR physical therapy modalities OR rehabilitation.” The only secondary term used to search for articles on pharmacotherapy was “duloxetine,” as that is the only medication formally approved in some countries for treatment of SUI. For identifying publications on transurethral radiofrequency collagen denaturation, the secondary terms “Renessa OR radiofrequency OR radiofrequency collagen denaturation OR radiofrequency micro-remodeling OR electromagnetic fields” were used. Searches to identify trials of bulking agents were limited to products approved by the Food and Drug Administration for treatment of SUI. Secondary search terms were “injection OR collagen OR biocompatible materials OR bulking agents OR Contigen OR Durasphere OR Uryx OR Coaptite OR Macroplastique.” 

#### 2.1.1. Supplemental Searches

An additional search of PubMed to identify potential articles was conducted using the secondary terms “systematic review OR meta-analysis.” A search of the Cochrane Library was also conducted. The full-text versions of all relevant articles were obtained, and the bibliographies were manually reviewed for additional eligible study reports. 

### 2.2. Eligibility Criteria and Study Selection

The eligibility of each study was assessed based on search results. Studies were eligible for inclusion if they were prospectively designed, all patients were females aged 18 years or older, and the study duration was at least 12 months after initiation of treatment. To gain a historical perspective of the long-term effectiveness of pelvic floor rehabilitation, this search was originally limited to studies describing a comparison of therapies or comparison of a therapy versus placebo or no treatment, and employing a randomized or quasirandomized (i.e., allocation by alternation) design. Relevant study designs were required to define PFMT as a regimen wherein the proper procedure for performing pelvic floor muscle contractions was taught by a health care professional, and the exercises were performed by patients in repeated sets on a regular basis. Following the original searches, however, it was discovered that few randomized, controlled, blinded trials of at least 12 months in duration existed in the literature, and the criteria for inclusion were adjusted to permit the assessment of the best available evidence for each treatment. 

Exclusion criteria included articles reporting on case reviews, studies of urinary incontinence related to significant factors outside the urinary tract (e.g., neurologic disorders, cognitive impairment, lack of independent mobility), nocturnal enuresis, studies that recruited men (e.g., SUI following prostatectomy), studies that included antenatal or postnatal women (within 3 months of delivery), bladder training studies, and studies that included patients with urge incontinence and did not distinguish between subgroups adequately to identify SUI data. Additionally, studies of therapies that are no longer marketed or that are not approved for treatment of SUI were excluded. 

## 3. Results

**Figure 1:**
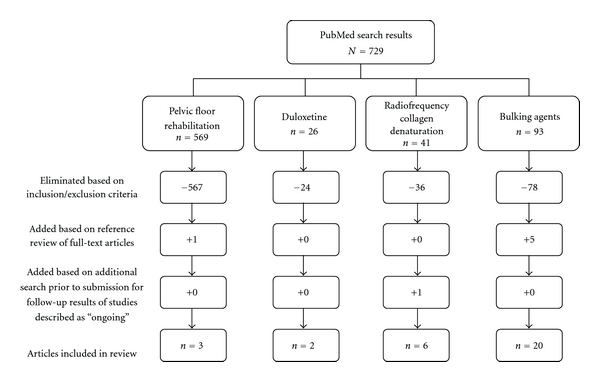
Search results and article selection.

The primary literature search yielded 729 potential publications. Additionally, 6 articles revealed during manual scans of the bibliographies of 14 systematic reviews found during supplemental searches of PubMed [5, 9, 20–23] and the Cochrane Library [24–31] were considered to be potentially eligible. In total, 31 studies involving 7038 patients with SUI met the inclusion criteria.  Figure 1 illustrates the details of each search process. Eligible publications included 3 articles on pelvic floor rehabilitation, 2 on duloxetine pharmacotherapy, 6 on transurethral collagen denaturation, and 20 on bulking agent treatments. Efficacy outcomes most commonly used in these trials were “cured,” defined as complete absence of incontinence episodes, and “improved,” defined as a reduction from baseline in incontinence grade or severity. Several studies also used the terms “continent” or “dry,” with definitions varying between no further SUI leaks and leakage less than a prespecified amount. Other outcome measures often included the number of incontinence pads used per day and the frequency of SUI episodes. 

### 3.1. Long-Term Results from Pelvic Floor Rehabilitation

As summarized in Table 1, the literature search identified three long-term prospective trials of pelvic floor rehabilitation involving 694 patients, with mean ages ranging from 31 to 69 years [32–34]. In these trials, PFMT regimens varied widely. All three trials had a 12-month end point, and patients were randomized to treatment groups. In one trial, all patients performed 20 pelvic floor muscle contractions 5 times daily. One group performed PFMT alone, while three groups received either PFMT therapy with reminders (stickers), PFMT with reminders and patient education, or PFMT with reminders, patient education, and counseling by a physiotherapist [32]. In another trial, after 12 weeks of training with a physiotherapist, patients completed 20-minute at-home sessions of PFMT with biofeedback 5 days per week. One group used a personal biofeedback monitor device [33]. In the final study, at-home Kegel exercises were performed 5 times daily, eventually decreasing to twice daily, with patients also receiving either eleven 30-minute individual or nine 2-hour group sessions with a physiotherapist [34].

In all three studies, SUI symptoms showed significant improvement at 3 months that was maintained at final follow-up in most patients. Improvement was shown in patient-reported SUI symptoms in two of the studies [32, 34] and, in the other, in electromyographically (EMG) assessed muscle electrical activity [33]. In all three trials, PFMT efficacy was comparable, regardless of whether it was performed alone versus in a group [34], with patient education [32], or paired with at-home biofeedback [33]. The positive outcomes reported in one trial [33], however, are difficult to interpret because findings were reported separately for patients who failed therapy versus those with treatment success. In the trial conducted by Aukee et al., 14 of 35 patients (40%) had undergone or were scheduled for incontinence surgery during long-term follow-up, while patients without surgery who adhered to therapy continued to show positive outcomes (Table 1). 

Safety and tolerability were not described in these reports. In the trial by Aukee et al. [33], the EMG-assisted biofeedback device was described as “not harmful” by patients, and no adverse events with device use were reported. 

### 3.2. Pharmacotherapy

The search yielded no randomized controlled trials of duloxetine (Yentreve, AriClaim; Eli Lilly, Indianapolis, IN) with a study duration of at least 12 months. The search did reveal results from two long-term, open-label trials of duloxetine therapy for SUI, which are described in Table 2 [35, 36]. These trials assessed a total of 4167 patients who took duloxetine at the recommended dosage of 40 mg bid for up to 30 months. Across these trials, patient discontinuation rates ranged from 57.5% to 91%. In the trial by Vella et al., most women discontinued duloxetine therapy within 4 weeks of initiation, citing side effects (45.2%) or perceived lack of efficacy (23.7%) as the primary reason for discontinuation [35]. In the study by Bump et al. [36], 57.4% to 71.5% of women who continued with long-term duloxetine therapy at end point reported that their SUI symptoms were “much” or “very much” improved. In line with this, a subset of these women throughout follow-up and at end point reported an approximate 60% to 70% reduction in the frequency of incontinence episodes. These reports described neither the nature of common adverse events with duloxetine nor adverse events leading to treatment discontinuation. 

### 3.3. Transurethral Radiofrequency Collagen Denaturation Studies

Although bulking agents and transurethral radiofrequency collagen denaturation (Renessa; Novasys Medical, Inc., Newark, CA) are both nonsurgical treatment options for women with SUI, they are indicated for use in different patient populations. The one-time radiofrequency collagen denaturation procedure requires no incisions and can be performed with the use of local anesthesia in a physician's office or outpatient treatment center. The procedure utilizes a probe shaft with a balloon tip to deliver low levels of radiofrequency energy at 65°C, creating localized microscopic collagen denaturation sites within the bladder neck and proximal urethra without creating strictures, fibrosis, significant tissue necrosis, gross shrinkage, or neurovascular injury. The denatured collagen results in reduced dynamic compliance of the surrounding untreated tissue [17, 37]. Following the procedure, the patient typically voids and leaves the treatment site within an hour. 

As summarized in Table 3, five trials of transurethral radiofrequency collagen denaturation (*N* = 371) [37–41] were identified by the original literature search. An additional search prior to publication for articles detailing follow-up results from an ongoing study revealed one new article [42]. The longest follow-up period described was 3 years [40]. An early 12-month study by Sotomayor and Bernal [37] examined the use of four different timing and placement methods for the delivery of radiofrequency energy (e.g., 60-second versus 90-second increments). Cure rates ranged from 22% to 67%, with reductions in episode frequency from 67% to 89% of patients. In a later randomized, controlled trial [41], leak point pressure was significantly improved with radiofrequency collagen denaturation versus an identically performed sham treatment at 12 months posttreatment. Furthermore, patients with moderate to severe SUI demonstrated a significant improvement in quality of life compared with the sham treatment arm (*P* = .04). One retrospective analysis of this trial found that outcomes did not differ based on menopausal status and use of hormonal replacement therapy [38]. The long-term durability of this treatment was reported in a 3-year follow-up study by Appell et al. that found that 56% of women who underwent radiofrequency collagen denaturation continued to report a 50% or greater reduction in SUI episode frequency [40]. 

In a report by Elser et al., 12-month results from an ongoing 36-month study showed that 45% of patients were considered dry, with 29% experiencing no leaks and 16% leaking less than 1 gram on a standardized in-office pad weight test. Additionally, 69% of women experienced a 50% or greater reduction in leakage, and 71% reported improvements on quality of life measures. A follow-up report from the same study revealed that 61.7% of evaluated patients continued to experience at least a 50% reduction in SUI leaks at 18 months [42]. These studies showed radiofrequency collagen denaturation to be generally safe and well tolerated. Overall, adverse events were transient and mild, including dysuria, urgency, and urinary tract infection, with no serious adverse events reported. 

### 3.4. Bulking Agents

Women with SUI due to ISD may elect to undergo nonsurgical treatment with a bulking agent. As with transurethral collagen denaturation, these treatments may be administered in a physician's office or outpatient treatment center with the use of local anesthesia. The agents are injected through a needle placed transurethrally through a urethroscope or periurethrally lateral to the urethra while observing the proximal urethra through a urethroscope; they work by augmenting the urethral sphincter. Typically, patients may leave the treatment site about 1 hour following treatment. 

The literature search revealed 20 trials meeting the inclusion criteria that evaluated five types of bulking agents (Table 4). Among them, nine investigations (*N* = 682) [19, 43–50] described the long-term efficacy of glutaraldehyde cross-linked (GAX) collagen (Contigen; Bard, Covington, GA). Patients typically received an initial series of 1 to 4 transurethral injections over several months, as needed. Rates of patients deemed cured or improved ranged from 21% to 81% at 12 months, 7% to 52% at 2 years, and 30% to 43% at more than 4 years. Notably, however, the studies with end points of more than 4 years reported that patients required repeated collagen injections during the follow-up period to restore improvement [19, 46]. Similarly, two long-term trials [51, 52] (*N* = 321) of carbon-coated zirconium beads (Durasphere; Carbon Medical Technologies, St. Paul, MN) reported cure or improvement rates between 35% and 80% at 1 year, but efficacy at 3 years [51] declined, with only 21% of patients reporting improvement. Adverse events with these agents included urinary urgency, transient urinary retention, difficulty voiding, and urinary tract infection.

Among eight long-term trials (*N* = 507) [54–61] of cross-linked polydimethylsiloxane injections (Macroplastique; Uroplasty, Minnetonka, MN), cure rates at 12 months posttreatment ranged between 20% and 71%, with improvement rates between 19% and 48% (Table 4). With follow-up extending up to 60 months, cure rates ranged from 18% to 40% and improvement rates ranged between 33% and 39%, with repeated injections required to maintain efficacy [59, 61]. A study of the injectable agent calcium hydroxylapatite (Coaptite; Boston Scientific, Natick, MA; *N* = 296) [62] showed similar long-term results. Reported adverse events included transient urinary retention, urgency, dysuria, and urinary tract infections. 

## 4. Discussion

While randomized controlled trials are generally considered to be the best evidence, it appears that it may be difficult to conduct such trials in women with SUI, most likely because each treatment involves different methods, making blinding difficult. Also, treatments such as transurethral radiofrequency collagen denaturation and bulking agents are indicated for use in different patient populations, making head-to-head comparisons implausible. Thus the best available evidence was often found in open-label studies of women with SUI, and search results of pelvic floor rehabilitation, pharmacotherapy, transurethral radiofrequency collagen denaturation, and bulking agents were permitted to include open-label studies that met the other eligibility criteria.

A surprising find of this literature review was the paucity of prospectively designed studies of pelvic floor rehabilitation of at least 12 months in duration, even though PFMT has been used for treating patients with SUI since the 1950s [63, 64]. While some follow-up results were available for up to 10 years or so following treatment initiation, the original prospectively designed trials were no longer than 6 months in duration, leaving only 3 articles eligible for inclusion in this paper. 

Despite the paucity of prospective long-term trials, noninvasive therapies continue to be widely used as first-line therapy [5, 9]. There are no or minimal associated safety risks, the costs are low, and the short-term benefits—seen in approximately 60% to 77% of patients—are well established [3, 8, 9, 65–67]. Lasting improvements, however, are highly dependent on ongoing patient compliance. Pelvic floor rehabilitation can yield benefits that persist in some patients for at least 12 months. However, for many patients (up to 40% in one study [33]), PFMT failed within 1 year of initiation, possibly indicating lack of adherence to these therapies. 

Similarly, large numbers of patients prescribed duloxetine discontinued within the first month, primarily because of adverse events or lack of efficacy. 

Transurethral radiofrequency collagen denaturation and urethral bulking agents offer consistent benefits that tended to persist for at least 12 months for many patients. Results with this procedure demonstrated that more than 50% of patients continued to experience improvements in SUI symptoms up to 3 years following a single treatment. This is in contrast to results with urethral bulking agents, indicated for treatment of patients with SUI due to ISD. With longer follow-up, the benefit seen with bulking agents diminished and patients typically required additional injections. Moreover, the reported persistence of response to a single injection was, in some cases, difficult to distinguish from the effect of repeated injections. Additionally, the clinician should be experienced in the technical aspects of the procedure to optimize outcomes. 

The primary goals of SUI treatment include safety, efficacy, and durability. In the present paper, pelvic floor rehabilitation, with or without adjunctive therapy, was shown to be an effective and safe first-line treatment for the majority of female SUI patients, at least in the short-term. Short-term data indicate that many women will initially respond well to pelvic floor rehabilitation [65–67]. Moreover, for patients who persist with the technique over time, improvements can be maintained for 1 year or longer, but higher-quality, longer-term outcomes data with PFMT are needed. Although no long-term advantage of adjunct therapy with PFMT was described in the randomized, controlled studies examined herein, an observational study conducted over 60 months found that combination therapy of PFMT with vaginal weights, electrical stimulation, and/or biofeedback was successful [68]. In patients with significant urethral sphincteric denervation injury and resultant intrinsic deficiency, however, bulking agents or tight slings may represent the only appropriate options besides a radical diversion procedure.

While no long-term studies of weighted vaginal cones were found, short-term studies report treatment success rates from 70% to 80% for up to 6 months [69, 70]. Additionally, an observational trial by Komesu et al. found that the use of pessaries was effective in patients for up to 12 months [71]. New products, such as the intravaginal polycarbonate sphere (Colpexin Sphere, Adamed Inc., Rutherford, NJ), may also be useful in patients with SUI but require controlled investigation. Efficacy data available to date are limited to use in women with pelvic organ prolapse [10]. 

Although the studies reviewed herein revealed no adverse effects associated with pelvic floor rehabilitation, treatment-emergent adverse events have been reported with the use of electrical stimulation and vaginal cones [72, 73]. Minor bleeding and vaginitis have occurred with the use of vaginal cones [72], while adverse events reported with electrical stimulation included vaginal irritation, vaginal infection, and occasional episodes of pain [72, 73]. No serious or irreversible events were reported.

No pharmacologic treatment has been approved by the US Food and Drug Administration for treatment of SUI, but duloxetine is approved in many countries and is sometimes prescribed by US physicians for SUI patients. While hormone therapy is also sometimes prescribed off-label as pharmacotherapy for women with SUI, three long-term randomized controlled trials found that this therapy is not consistently effective. One 2-year trial of daily oral estriol combined with PFMT versus PFMT alone found significantly improved SUI scores in the estriol group (*P* < .0001) for up to 12 months [74]. However, a 2-year study of transdermal ultra low-dose estradiol found no significant differences in the reduction of SUI symptoms versus placebo [75], and a 4-year study of daily oral estrogen plus progestin found that incontinence improved in only 20.9% of patients and worsened in 38.8% of patients [76]. Importantly, oral hormone therapy has been associated with cardiovascular risks, in particular the risk of venous thromboembolism, in postmenopausal women [77–79]. Although a recent study indicated that transdermal hormone therapy may exhibit a better safety profile with regard to thrombotic risk [79], the effects are not fully established and the benefits should be weighed against the risks for individual patients [78, 79]. 

Among patients for whom nondrug and pharmacologic conservative therapy fails, surgical intervention is usually recommended. As an alternative to surgery, female patients with SUI may consider nonsurgical procedures, including urethral bulking agent injections or transurethral radiofrequency collagen denaturation, depending on whether they are diagnosed with SUI associated with ISD or with SUI associated with urethral hypermobility. Because of these distinctions, careful pretreatment patient evaluation is necessary; selecting the proper type of technique for a given patient may help to optimize outcomes with such procedures. 

With radiofrequency collagen denaturation, the reviewed studies indicate that a single treatment procedure is associated with lasting SUI improvements, in one trial persisting in 56% of patients for up to 3 years [40]. This compares positively with results of multiple injections with bulking agents for patients with SUI and with some of the more recent surgical results. One analysis indicated improved QOL at 1 year posttreatment [38]. Safety was described as good, with the most common adverse events including dysuria and urinary tract infection; no serious complications with this procedure have been reported. 

It is important to distinguish this procedure from an older radiofrequency procedure, transvaginal radiofrequency tissue ablation (SURx; Cooper Surgical, Trumbull, CT). With transvaginal tissue ablation, an incision was made through the vagina, lateral to the urethra, and the vaginal mucosa was dissected off the underlying endopelvic fascia to expose the surface area for treatment. Radiofrequency energy was applied at high temperatures (85°C) to large areas of tissue, causing blanching and gross shrinkage secondary to scarring and coagulation necrosis of the affected tissue [80, 81]. Tissue damage likely resulted in nerve ending damage and the development of worsening urethral sphincteric function due to partial denervation. The procedure was typically performed in a hospital setting under general anesthesia, but is no longer marketed following reports of low cure rates and the frequent need for additional corrective treatment [82–84]. 

In distinct contrast, transurethral collagen denaturation requires no incision, and radiofrequency energy at low temperatures of 65°C is applied to very small areas of submucosal tissue at much lower temperatures, resulting in tissue remodeling rather than gross tissue shrinkage or necrosis. 

The present review of the literature indicates that long-term outcomes vary among available bulking agents. GAX collagen appears to offer less durable improvements, more often requiring repeat injections, compared with the newer silicone or polymer-based agents, which are considered more permanent. In the longest follow-up studies of such urethral bulking agents [59, 61], cure rates ranged from 18% to 45% and improvement rates ranged between 33% and 39% at 50 to 60 months posttreatment. Safety with these products appears to be generally good, although serious complications rarely occur, such as migration of the bulking material into other tissues and urethral erosion [85, 86]. The literature search revealed one long-term study of the injectable agent ethylene vinyl alcohol (Tegress; Bard, Covington, GA; *N* = 33) [87], but this agent was found to have significant adverse effects, such as tissue necrosis, and is no longer marketed. It appears that the ideal bulking agent has not yet been identified. Such an agent would be inert, long-lasting, nonmigrating, adjustable, and easy to inject. 

### 4.1. Limitations

The present paper has some limitations that must be considered. One difficulty found in this literature review is the lack of standardized methodology as well as varying terminology used in SUI studies, making it problematic to directly compare results. Among the studies included, there is a variability in design, baseline patient characteristics, specifically with regard to the type of incontinence, the method of diagnosing SUI, and the severity of symptoms. This could account for the variability in outcomes and the range in success rates between different studies of similar therapies. Such disparities have been reported before [88–90], suggesting an important need to apply standardized terminology and methods to all studies of SUI.

The PFMT regimens employed also varied widely, making cross-study comparisons of outcomes difficult to interpret. Moreover, across all types of therapies reviewed currently, SUI efficacy end points differed substantially. Finally, no head-to-head comparisons of different classes of nonsurgical techniques were reviewed in this study (e.g., PFMT versus a bulking agent; a bulking agent versus radiofrequency collagen denaturation). Head-to-head randomized controlled studies of nonsurgical therapies for patients with SUI are difficult to design primarily because each treatment is approved for use in different patient populations, making comparison of results between treatment groups difficult. 

A common problem that exists among long-term SUI studies is the patient dropout rate. While it is known that some patients go on to have surgery, others may decide they no longer have time or desire to participate in follow-up visits over several years. As a result, the patient population in long-term SUI studies may diminish over time, making it difficult to compare results. Another common discrepancy that occurs when comparing study results for any SUI treatment was highlighted in a recent clinical study. A 24-month study by Albo et al. [91] found much lower cure rates for the Burch colposuspension (47%) and fascial sling (38%) procedures than those commonly reported in the literature (up to 90%). The differences in success rates were most likely attributable to the stricter definition of cured used in the study compared with varying definitions of cured or dry used in previous studies. Such variability in outcomes definitions exists in studies of all SUI treatments, not just surgeries, and can make it difficult to compare results between studies [88–90, 92, 93].

## 5. Conclusions

While pelvic floor rehabilitation is generally the first-line treatment for female patients with SUI, the long-term benefits are unclear and further clinical studies of at least 12 months in duration are needed. This literature analysis suggests that minimally invasive, nonsurgical transurethral radiofrequency collagen denaturation is an effective option for patients with SUI due to urethral hypermobility who have failed pelvic floor rehabilitation and who cannot or choose not to undergo surgery. Evidence suggests generally good safety and benefits that persist for at least 3 years for the majority of patients who undergo this procedure. Bulking agents used for patients with SUI due to ISD have also demonstrated effectiveness at 1 year, but results, particularly with older agents, tend to diminish upon further follow-up; however, improvement may be enhanced through repeated injections. Each of these minimally invasive options appears to present an intermediate management step after failure of conservative therapy but prior to surgery. 

## Figures and Tables

**Table 1:** Summary of conservative therapy trials.

Study	*N*	Age, y	Treatment	Study design	Study duration	Efficacy measures	Efficacy outcomes
Alewijnse et al. 2003 [32]	129	Mean, 55.6	PFMT alone (control; *n* = 32) PFMT + reminders (*n* = 29) PFMT + reminder + patient education guide (*n* = 34) PFMT + reminder + patient education guide + counseling (*n* = 34)	Randomized, controlled, open label	12 mos	Self-administered questionnaires; diaries	Overall 75% cured or improved at 12 mos Overall reduction in number of SUI episodes in all groups from mean 23/wk to 8/wk; *P* < .001 Efficacy between all treatment groups, *P* = NS

Aukee et al. 2004 [33]	35	Mean, 49.4, 51.4 Range, 31–69	PFMT alone (control; *n* = 19) PFMT+ biofeedback, with at-home EMG training device (*n* = 16)	Randomized, controlled, open label	12 mos	EMG-evaluated pelvic floor muscle activity; need for surgical intervention after conservative treatment; patient assessment using leakage index (based on 13 types of physical exertion that may trigger SUI episodes)	Nonoperated EMG results: PFMT, *P* = .02 PFMT+ biofeedback, *P* = .005 Treatment failure (surgery), *n* = 14 Only nonoperated biofeedback group achieved significant reduction in leakage index, *P* = .005

Janssen et al. 2001 [34]	530	Mean, 47.8	PFMT, group sessions (*n* = 404) PFMT, individual sessions (*n* = 126)	Randomized, open label	12	Written questionnaires; diaries; patient exercise forms	Reduction in number of SUI episodes versus baseline: Group, *P* < .001 Individual, *P* < .001 Group versus individual, *P* = NS

Abbreviations: EMG, electromyography; NS, not significant; PFMT, pelvic floor muscle training; SUI, stress urinary incontinence.

**Table 2:** Summary of pharmacotherapy trials.

Study	*N*	Age, y	Treatment	Study design	Study duration	Efficacy measures	Efficacy outcomes
Bump et al. 2008 [36]	Cohort A: 1424 Cohort B: 2515	Cohort A: 53.2 Cohort B: 55.5	Duloxetine 40 mg bid	Pooled analysis Cohort A: 3 randomized, placebo-controlled, double-blind studies and 1 open-label, single arm trial Cohort B: one randomized, placebo-controlled, double-blind study	30 mos 18 mos	Cohort A: PGI-I scores; discontinuation rates Cohort B: PGI-I scores; diaries; discontinuation rates	PGI-I much or very much better: Cohort A: 71.5% Cohort B: 57.4% Decrease in SUI episodes at end point: Cohort A: N/A Cohort B: *≈*70% Discontinued by 12 months: Cohort A: 57.5% Cohort B: 79.5%

Vella et al. 2008 [35]	228	N/A	Duloxetine 40 mg bid	Open label, single arm	12 mos	Discontinuation rates	Discontinued by 4 wks: 69% Discontinued by 12 mos: 91% At 12 mos, 10 women were on no treatment and had not undergone surgery; of these, 6 were considered cured

Abbreviations: N/A, not available; PGI-I, Patient Global Impression-Improvement rating scale.

**Table 3:** Summary of transurethral radiofrequency collagen denaturation trials.

Study	*N*	Age, y	Treatment	Study design	Study duration	Efficacy measures	Efficacy outcomes
Appell et al. 2006 [41]	173	Mean, 50 Range, 22–76	One-time radiofrequency collagen denaturation (*n* = 110) Sham procedure (*n* = 63)	Randomized, sham-controlled, single blind	12 mos	I-QOL scores; LPP testing	≥10-point I-QOL improvement: Treated patients: 48% Sham: 44% Mean LPP: Treated patients: 13.2 ± 39.2 cmH_2_O increase versus Sham patients: −2.0 ± 33.8 cmH_2_O; *P* = .02

Appell et al. 2007 [40]	21	Mean, 52.2 Range, 39.0–65.4	One-time radiofrequency collagen denaturation	Retrospective follow-up of a 12-month trial	36 mos	Diaries; I-QOL scores	Mean I-QOL scores improved 12.7 points ≥50% decrease in SUI episodes: 56%

Elser et al. 2009 [39]	136	Mean, 47 Range, 26–87	One-time radiofrequency collagen denaturation	Open label, single arm, interim results	12 mos	Diaries; in-office stress pad weight tests; I-QOL scores; UDI-6 scores; PGI-I scores	≥50% decrease in SUI episodes: 50% ≥50% reduction in leakage: 69%; 45% of patients were dry Mean I-QOL scores improved 9.5 points Mean UDI-6 scores improved 14.1 points PGI-I “a little,” “much,” or “very much” better: 50%

Elser et al. 2010 [42]	136^a^	Mean, 47 Range, 26–87	One-time radiofrequency collagen denaturation	Open label, single arm, interim results	18 mos	Diaries; I-QOL scores; UDI-6 scores; PGI-I scores	≥50% decrease in SUI episodes: 47% Mean I-QOL scores improved 10.9 points Mean UDI-6 scores improved 13.0 points PGI-I “a little,” “much,” or “very much” better: 47%

Lenihan et al. 2005 [38]	73^b^ women in a subgroup with baseline moderate to severe SUI	N/A	One-time radiofrequency collagen denaturation (*n* = 43) Sham procedure (*n* = 30)	Post hoc analysis of a randomized, sham-controlled, single-blind study	12 mos	I-QOL scores	≥10-point I-QOL improvement: Treated patients: 74% Sham: 50% *P* = .03

Sotomayor and Bernal 2005 [37]	41	Mean, 47.6 Range, 34–81	One-time radiofrequency collagen denaturation, with varying submucosal targets (4 subgroups)	Quasirandomized	12 mos	I-QOL scores; physician-administered questionnaire	Mean I-QOL scores improved 16–24 points ≥50% decrease in SUI episodes: 63%–89% Cured: 22%–67%

Abbreviations: I-QOL, Incontinence Quality of Life questionnaire; LPP, leak point pressure; PGI-I, Patient Global Impression of Improvement; SUI, stress urinary incontinence; UDI-6, Urogenital Distress Inventory.

^a^These patients were already counted in Elser et al. 2009 [39], thus they were not included in the total patient numbers as given in the text.

^b^These patients were already counted in Appell et al. 2006 [41], thus they were not included in the total patient numbers as given in the text.

**Table 4:** Summary of included bulking agent trials.

Study	*N*	Age, y	Treatment	Study design	Study duration	Efficacy measures	Efficacy outcomes
Glutaraldehyde cross-linked (GAX) collagen							

Bent et al. 2001 [50]	90	Mean, 60.9 Range, 35–86	Up to 3 injections, as needed, over 6 mos	Open label, single arm	12 mos	Continence grade; diaries; LPP testing, QOL questionnaire	Dry: 21% Improved: 21% Improved LPP: 54% QOL improvement: 62% ≥1 continence grade improvement: 21%

Corcos and Fournier 1999 [19]	40	Mean, 2.3 Range, 38–82	Up to 5 injections, as needed	Open label, single arm	Mean follow-up, 50 mos	Direct patient questioning; PVR test; stress pad weight test; Valsalva LPP	Cured: 30% Improved: 40% All cured patients had PVR <10 mL and negative pad tests

Elsergany et al. 1998 [43]	33	Mean, 64 Range, 19–97	Up to 3 injections, as needed	Open label, single arm	Mean follow-up, 18.8 mos	Stamey Urinary Incontinence Scale; daily pad usage; diaries	Cured: 48.5% ≥1 Stamey Scale grade improvement: 33.3% Improved from baseline, all *P* < .05

Herschorn et al. 1996 [44]	187	Mean, 62.9 Range, 15–94	Up to 3 injections	Open label, single arm	Mean follow-up, 22 mos	Direct patient questioning	Cured: 23% Improved: 52%

Homma et al. 1996 [45]	78	Mean, 57.1–63.5	Injections as needed	Open label, single arm	24 mos	Patient-administered questionnaire	No SUI leaks: 6.7% SUI episodes daily: 50% <1 SUI episode/wk: 16.7% <1 SUI episode/day: 26.7% Mean injections, 1.9

Monga et al. 1995 [53]	60	Mean, 64 Range, 20–90	Up to 3 injections	Open label, single arm	24 mos	Direct patient questioning; cystometry; stress pad weight test	Patient-rated cured at 3, 12, 24 mos: 46%, 40%, 48%; improved: 40%, 37%, 20% Objective measures (cystometry/pad weight), cured at 3, 12, 24 mos: 61%, 54%, 48% Mean injections: 1.6

Richardson et al. 1995 [46]	42	Mean, 64 Range, 28–88	1–8 injections, as needed	Open label, single arm	Mean follow-up, 46 mos	LPP testing; incontinence grades (0–3)	Cured: 40% Greatly improved/improved: 43% Unchanged/worse: 17% LPP improvement in women cured/greatly improved: 65.4 cmH_2_O Mean injections: 2.4 in those cured/greatly improved; 4.1 in those improved/unchanged/worse

Smith et al. 1997 [47]	94	67	Injections as needed	Open label, single arm	≥18 mos	Patient self-report	Initially dry/improved: 67% Of these, 66.7% still dry/improved at 18 mos; most continent with 1–3 injections, 7 required 4 or more

Winters et al. 2000 [48]	58	Mean, 73.2 Range, 65–86	Injections as needed	Open label, single arm	24 mos	Telephone interview	Maximal/moderate improvement: 62.5% Maximal/moderate QOL improvement: 45.0% Mean injections: 1.9 Mean time to SUI recurrence: 7.9 months

Carbon-coated zirconium beads							

Chrouser et al. 2004 [51]	86	Mean, 67	Carbon-coated zirconium beads (*n* = 43) GAX collagen (*n* = 43) Injections as needed	Post hoc, open label versus GAX collagen (control)	36 mos	Patient questionnaire	SUI improvement at 12, 24, 26 mos: Zirconium beads: 35%, 33%, 21% GAX collagen: 33%, 19%, 9% Overall, *P* = NS Mean injections: N/A

Lightner et al. 2001 [52]	235	Mean, 57.7 Mean, 57.0 Range, 26–84	Carbon-coated zirconium beads (*n* = 115) GAX collagen (*n* = 120) Maximum 5 injections, as needed	Randomized, controlled, single blind versus GAX collagen (control)	12 mos	Stamey Urinary Incontinence Scale; stress pad weight test	≥1 Stamey Scale grade improvement: Zirconium beads: 66.1% GAX collagen: 65.8% *P* = NS Mean change in pad weight: Zirconium beads: 27.9 g GAX collagen: 26.4 *P* = NS Mean injections: 1.69 versus 1.55

Polydimethylsiloxane							

Ghoniem et al. 2009 [54]	247	Mean, 61	Polydimethylsiloxane (*n* = 122) GAX collagen (*n* = 125) Injections as needed	Randomized, single blind versus GAX collagen	12 mos	Stamey Urinary Incontinence Scale; stress pad weight test; I-QOL scores	≥1 Stamey Scale grade improvement: Polydimethylsiloxane: 61.5% GAX collagen: 48.0% I-QOL improvement: Polydimethylsiloxane: 28.7% GAX collagen: 26.4% Pad weight test, *P* = NS

Koelbl et al. 1998 [55]	32	Mean, 64.3 Range, 39–85	Up to 2 injections	Open label, single arm	12 mos	Stamey Urinary Incontinence Scale; cough stress test; urethral pressure measurements	Cured: 60% Increased maximal urethral pressure, *P* = .03

Maher et al. 2005 [56]	45	Mean, 65 Range, 34–84 Mean, 63 Range, 43–81	Polydimethylsiloxane (*n* = 23) Pubovaginal sling (*n* = 22)	Randomized, controlled	60 months	Patient questionnaire	Response rate, 60% in both groups Cured/improved: Injection: 21% Sling: 69%

Radley et al. 2001 [57]	56	Mean, 53 Range, 26–81	Up to 3 injections	Open label, single arm	Mean follow-up, 19 months	Patient questionnaire	Cured/improved: 19.6% Improved: 39.3% No longer using pads: 19.6%

Tamanini et al. 2003 [58]	21	Median, 47.4 Range, 33–54	Injections as needed	Open-label, single arm, interim results	12 mos	Stamey Urinary Incontinence Scale; King's Health Questionnaire; pad usage; LPP testing; stress pad weight test	Cured: 76.2% Improved: 66.7% Mean decrease in daily pad usage: 3.1 Mean pad weight decrease: 45.7 g

Tamanini et al. 2006 [59]	21^a^	Median, 47.4 Range, 33–54	Injections as needed	Open label, single arm	60 mos	Stamey Urinary Incontinence Scale; King's Health Questionnaire; pad usage; LPP testing; stress pad weight test	Cured: 40.0% Improved: 33.3% Mean decrease in daily pad usage: 2.6 Mean pad weight decrease: 47.9 g
ter Meulen et al. 2009 [60]	45	Mean, 55 Range, 40–76	Up to 2 injections, as needed (*n* = 24) At-home PFMT (*n* = 21)	Randomized, controlled, open label versus PFMT (control)	12 mos	I-QOL scores; patient questionnaire; stress pad weight test	Polydimethylsiloxane: Cured: 88.9% Improved: 82.4% PFMT: 12-mo results N/A

Zullo et al. 2005 [61]	61	Mean, 69.7 Range, 55–82	One-time injection	Observational, open-label, single arm	60 mos	VAS scores; diaries, cough stress test; PVR; LPP	Cured: 18% Improved: 39% Cough stress test: Cured patients: negative Improved patients: positive only when standing Mean decrease in daily SUI episodes: 3.8 Mean changes in PVR and LPP: *P* = NS versus baseline

Calcium Hydroxylapatite							

Mayer et al. 2007 [62]	296	Mean, 61	Up to 5 injections, as needed	Randomized, single blind versus GAX collagen	12 mos	Stamey Urinary Incontinence Scale	≥1 Stamey Scale grade improvement: 63.4% (calcium Hydroxylapatite) versus 57.0% (GAX collagen); *P* = .34

Abbreviations: LPP, leak point pressure; N/A, not available; NS, not significant; PVR, postvoid residual; SUI, stress urinary incontinence; QOL, quality of life; VAS, visual analogue scale.

^a^These patients were already counted in Tamanini et al. 2003 [58], thus they were not included in the total patient numbers as given in the text.

## Abbreviations

EMG: Electromyography
GAX collagen: Glutaraldehyde cross-linked collagen
ISD: Intrinsic sphincter deficiency
PFMT: Pelvic floor muscle training
SUI: Stress urinary incontinence.
